# Comparative Morphophysiological Analyses and Molecular Profiling Reveal Pi-Efficient Strategies of a Traditional Rice Genotype

**DOI:** 10.3389/fpls.2015.01184

**Published:** 2016-01-05

**Authors:** Poonam Mehra, Bipin K. Pandey, Jitender Giri

**Affiliations:** Plant Nutrition Laboratory, National Institute of Plant Genome ResearchNew Delhi, India

**Keywords:** metabolic flexibility, microarray, lipidomics, root system architecture (RSA), phosphate

## Abstract

Phosphate (Pi) deficiency severely affects crop yield. Modern high yielding rice genotypes are sensitive to Pi deficiency whereas traditional rice genotypes are naturally compatible with low Pi ecosystems. However, the underlying molecular mechanisms for low Pi tolerance in traditional genotypes remain largely elusive. To delineate the molecular mechanisms for low Pi tolerance, two contrasting rice genotypes, Dular (low Pi tolerant), and PB1 (low Pi sensitive), have been selected. Comparative morphophysiological, global transcriptome and lipidome analyses of root and shoot tissues of both genotypes grown under Pi deficient and sufficient conditions revealed potential low Pi tolerance mechanisms of the traditional genotype. Most of the genes associated with enhanced internal Pi utilization (phospholipid remobilization) and modulation of root system architecture (RSA) were highly induced in the traditional rice genotype, Dular. Higher reserves of phospholipids and greater accumulation of galactolipids under low Pi in Dular indicated it has more efficient Pi utilization. Furthermore, Dular also maintained greater root growth than PB1 under low Pi, resulting in larger root surface area due to increased lateral root density and root hair length. Genes involved in enhanced low Pi tolerance of the traditional genotype can be exploited to improve the low Pi tolerance of modern high yielding rice cultivars.

## Introduction

Phosphorus (P) is a critical element for plant growth and development. It is an essential component of nucleic acids, membrane lipids, and regulates many vital plant physiological processes like photosynthesis and respiration. Most of the natural soil P exists in the form of organic compounds or sparingly soluble cationic complexes. As a result, phosphate (Pi), an inorganic bioavailable form of phosphorus, is a limiting factor for ~67% of the world's cultivable soils (Gilbert, 2009). Modern agriculture relies intensively on high input of Pi-fertilizers to compensate for limited soil Pi. However, it has been predicted that at the current rate of extraction, global P reserves of rock phosphate will be depleted soon (www.ifdc.org). In addition, application of Pi fertilizers is not favored economically and environmentally. Ironically, less than 20% of the applied Pi is absorbed by plants whilst the remainder forms insoluble complexes and also runs-off into water bodies (Ha and Tran, 2013).

Several plant adaptive responses to low Pi have been described. These include modulation of Root System Architecture (RSA) for increasing topsoil foraging (Lynch, 2011; Pandey et al., 2013), enhancing the activity of Pi transporters for its uptake (Ai et al., 2009), secretion of organic acids and phosphatases to mobilize the Pi from soil organic/inorganic matter (Wang et al., 2011) and production of ribonucleases/lipases to remobilize the cellular Pi (Raghothama, 1999). Membranes in the form of phospholipids contain 15–20% of total organic P in a cell (Poirier et al., 1991). Under P starvation, membrane phospholipids are hydrolyzed, galactolipids which do not contain Pi are synthesized, and Pi is released as adaptive strategy (Nakamura, 2013). These adaptive responses are orchestrated by complex molecular networks involving several genes (Ha and Tran, 2013; Pant et al., 2015). A multi-component molecular network of Pi scavenging systems, the *Pho* regulon, has been studied widely in plants (Reviewed in López-Arredondo et al., 2014). This system employs transcription factors, ubiquitin ligases, miRNAs and several downstream genes to regulate the Pi homeostasis in plants (Rouached et al., 2010).

Cultivated rice genotypes can be classified as “Pi responsive” (higher yield under high Pi) and “Pi efficient” (yield protection under low Pi) (Gerloff, 1977). Most of the modern rice cultivars are “Pi responsive” as they had been developed and selected on soils supplemented with Pi fertilizers. These genotypes possess shallow root systems; well-adapted for enhanced Pi acquisition from the topsoil under high Pi conditions (Wang et al., 2010; Rose et al., 2011). They show efficient partitioning of photosynthates toward economic yield, contributing to their higher harvest index (HI). However, under low Pi conditions these modern genotypes exhibit severe yield losses and are of little value. In contrast, naturally existing low Pi tolerant genotypes including landraces and naturally inbred traditional cultivars have been cultivated on Pi poor soils for very long time and these genotypes possess the genetic and phenotypic competence to withstand low Pi conditions (Wissuwa and Ae, 2001). Therefore, traditional genotypes can be an excellent resource of genes to improve the low Pi tolerance of high yielding modern rice genotypes.

Global transcriptome analysis in response to Pi deficiency has been performed in Arabidopsis (Misson et al., 2005; Müller et al., 2007), rice (Wasaki et al., 2003, 2006; Pariasca-Tanaka et al., 2009; Li et al., 2010; Dai et al., 2012; Park et al., 2012; Cai et al., 2013; Oono et al., 2013; Secco et al., 2013), tomato (Wang et al., 2002), bean (Hernández et al., 2007), maize (Calderon-Vazquez et al., 2008), and mustard (Hammond et al., 2005). These studies have been proven extremely effective in unraveling many novel low Pi responsive genes. However, a comprehensive picture of low Pi tolerance mechanisms in naturally tolerant traditional genotypes is missing.

In the present study, we have profiled a modern low Pi sensitive rice genotype (PB1) and a traditional tolerant genotype (Dular) to investigate their differential behavior under low Pi. Our comparative morphophysiological, transcriptome and lipidome analyses of these genotypes revealed that the low Pi tolerance of Dular is likely due to efficient internal Pi remobilization and maintenance of root growth for Pi uptake under low Pi conditions. We also report the changes in phospholipid/galactolipids accumulation and their corresponding genes under low Pi. These Pi-efficient strategies of traditional genotypes can be exploited to improve the low Pi tolerance of high yielding modern rice genotypes.

## Materials and methods

### Plant material and growth conditions

Seeds of PB1 and Dular were surface-sterilized by 0.1% mercuric chloride for 15 min and thereafter, washed with sterile water five-times and germinated on wet filter paper for 2 days. Uniformly germinated seedlings were transferred to Pi sufficient (320 μM) and Pi deficient (1 μM) nutrient media (Yoshida et al., 1976) with iron supplemented as FeNaEDTA. Seedlings were grown in growth chamber with 16 h day (30°C)/8 h night (28°C) photoperiod, 250–300 μM photons/m^2^/sec photon density and 70% relative humidity. Containers filled with 15 liters nutrient solution were used to grow 30 seedlings per genotype per biological replicate. The nutrient solution (pH 5.5) was changed every 24 h. After 15 days, root and shoot tissues were harvested separately and immediately frozen in liquid nitrogen for further analyses. Soluble Pi estimation in roots and shoots and In-Gel APase assay were performed as described (Jain et al., 2007; Wang et al., 2011). Root and shoot lengths were measured manually using a ruler.

### Analysis of lateral roots and root angle

Seedlings were grown under low and sufficient Pi media in aseptic conditions using MS media with 0.2% phytagel for 15 days. For lateral root analysis, 15-day-old roots were imaged and lateral root length and density on primary roots were calculated using ImageJ 1.46r (http://imagej.nih.gov/ij). For the root angle study, seeds were transferred to the center of a basket containing soilrite which was pre-treated with 1 M HCl and repeatedly washed with Milli Q water. Dried soilrite was filled in a basket supported on a pot containing Yoshida media (+Pi and −Pi), pH 5.0−5.5. Media were refreshed after every 24 h. After 30 days, the numbers of roots emerging from bottom and sides of basket were counted manually to calculate the root angle.

### Extraction of total RNA

Total RNA was extracted from root and shoot tissues of 15-days-old seedlings using RNeasy Mini Kit (Qiagen) according to the manufacturer's instruction. Tissues from three seedlings were pooled together for isolation of a sufficient amount of total RNA. Purity and integrity of RNA was determined using Bioanalyzer (2100 Agilent technologies). RNA samples having RIN (RNA Integrity Number) value above 9.5 in root and 8 in shoot were used for microarray analysis.

### Affymetrix genechip hybridization, washing, and scanning

The differential gene expression in each tissue/genotype grown under low and sufficient Pi conditions was analyzed in three independent biological replicates using Affymetrix rice genome array (57 K) GeneChip® according to manufacturer's protocol. Washing and scanning was carried out at Affymetrix GeneChip® Fluidics Station 450 and GeneChip® Scanner 3000 7G, respectively. Probe cel intensities for each array were retrieved in .cel file format by Affymetrix® GeneChip® Command Console® Software (AGCC).

### Microarray data analysis

For microarray analysis, data from 24 .cel files representing root and shoot tissues of PB1 and Dular, raised under Pi deficient and sufficient conditions in three biological replicates were imported into “GeneSpring” software (Agilent Technologies Inc.). The normalization and probe intensities summarization were performed by GC-RMA. Three biological replicates of each sample, showing correlation coefficient value of ≥ 0.97 were considered for downstream analysis (Supplementary Table 1). To identify the “significantly expressed” probe sets, a Three-way ANOVA analysis with the following model was performed to dissect out the independent effects of all three main variables (Genotype, G; Tissue, T; and Phosphate, Pi) and their interactions:

(1) $$\begin{array}{l} {\text{Y}}_{\text{ijkt}}=\mu +{\text{T}}_{\text{i}}+{\text{G}}_{\text{j}}+{\text{Pi}}_{\text{k}}+({\text{T}}_{\text{i}}\times {\text{G}}_{\text{j}})+({\text{T}}_{\text{i}}\times {\text{Pi}}_{\text{k}})\\ \;+\;({\text{G}}_{\text{j}}\times {\text{Pi}}_{\text{k}})+({\text{T}}_{\text{i}}\times {\text{G}}_{\text{j}}\times {\text{Pi}}_{\text{k}})+{\text{e}}_{\text{ijkt}} \end{array}$$

Y_ijkt_ denotes random variable giving the response for observation t of the treatment at level i, j, k of Tissue, Genotype and Phosphate whereas e_ijkt_ is independent random variable. To decrease the number of false positives, Benjamini Hochberg correction was applied at *p*-value cut-off of ≤ 0.05. All subsequent analysis was carried out as described (Deveshwar et al., 2011).

Corresponding gene IDs for the final dataset were obtained from the Rice Oligonucleotide Array Database (http://www.ricearray.org/), KOME (http://cdna01.dna.affrc.go.jp/cDNA) and NCBI (http://www.ncbi.nlm.nih.gov). Probe sets showing ≥2-fold changes (FC) in expression under Pi deficiency in relation to their respective control tissue, were considered as “differentially expressed” (−Pi/+Pi). For evaluating relative genotypic effects (Dular/PB1), relative gene expression in Dular was calculated using PB1 as control. Finally, all significantly expressed genes were assigned annotations and GO terms using Rice Genome Annotation Database (http://rice.plantbiology.msu.edu/index.shtml). Differentially expressed genes were then searched in KEGG (http://www.genome.jp/kegg/pathway.html) and RiceCyc (http://pathway.gramene.org/gramene/ricecyc.shtml) for assigning their putative roles in cellular metabolism. Heat maps were generated in MeV (Multi Experiment Viewer) software, version 4.6.0. on log_2_ transformed FC and hierarchical clustering was done using distance matrix Pearson correlation.

Quantitative real time PCR (qRT-PCR) was performed in three replicates as described earlier (Deveshwar et al., 2011). Primers used for the qRT-PCR are listed in Supplementary Table 2.

### Lipid profiling by LC-QTOF

Plant lipids were extracted from 50 mg lyophilised samples of 15-day-old seedlings as described by Matyash et al. (2008). The Lipidome data were acquired using an Agilent 6530 QTOF for positive ion analysis and Agilent 6550 QTOF Mass spectrometer for negative ion analysis (employing Agilent jet stream thermal focussing technology). Raw data were processed by Agilent's Mass Hunter Qual software to find peaks. Peaks were then imported into Mass Profiler Professional for peak alignments and filtering. To annotate lipids, MSMS files were used to query the Lipid Blast library.

### Identification of DNA polymorphisms

SNPs and InDels in differentially regulated PSR genes were identified from whole genome resequencing data of both genotypes (Mehra et al., 2015). For identification of SNPs in promoters of key PSR genes, 2 Kb upstream promoter regions were analyzed.

## Results

### Comparative morphophysiological study revealed greater potential of dular to withstand low Pi conditions

**Figure 1:**
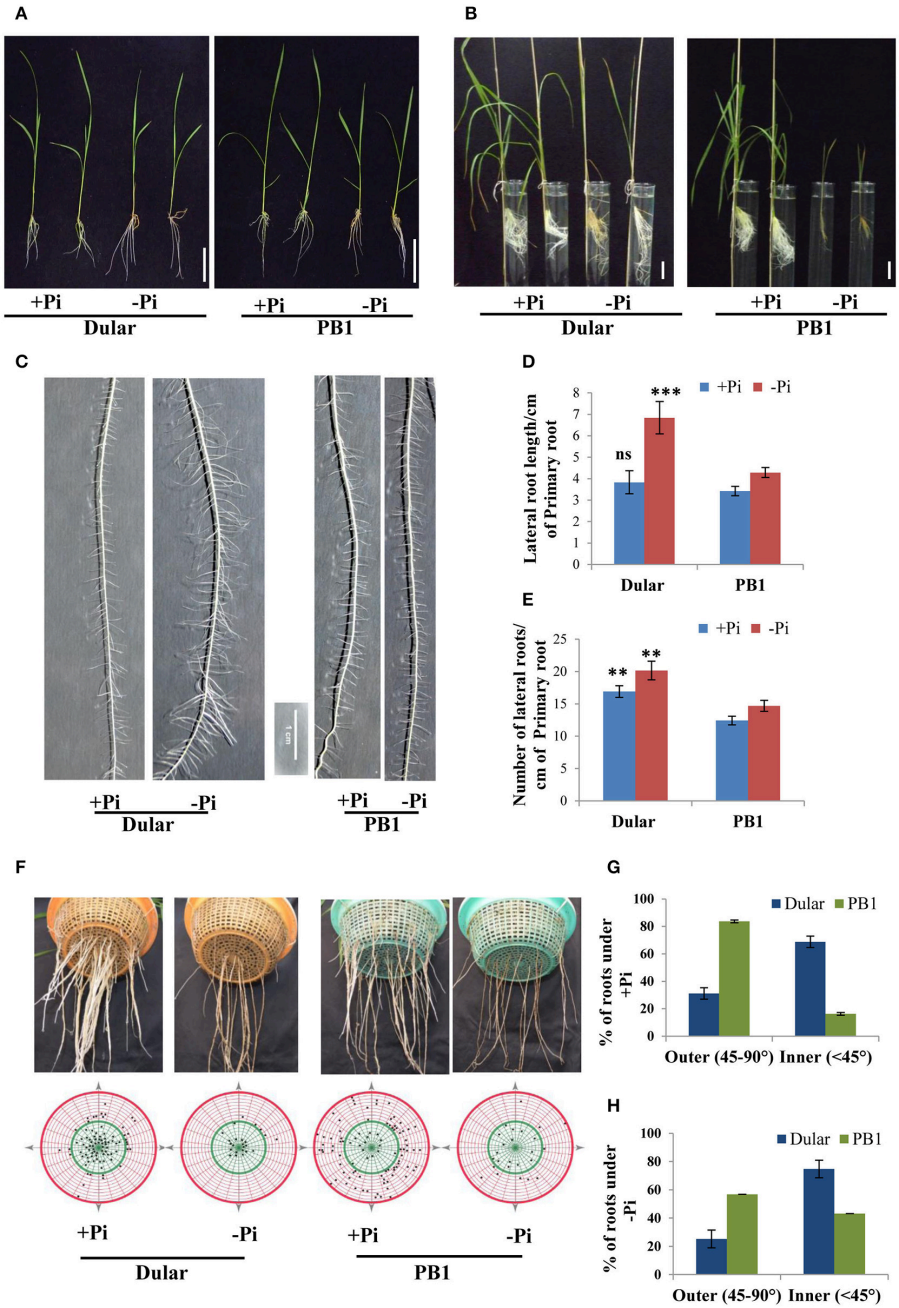
**Differential plant growth under Pi deficiency (A) Seedling growth of hydroponically grown Dular and PB1 raised under Pi sufficient (320 μM) and Pi deficient (1 μM) conditions in hydroponics after 15-days and (B) two-month**. **(C)** Lateral root phenotype; **(D)** length and **(E)** density of PB1 and Dular under +Pi and −Pi. **(F)** Root spread of PB1 and Dular under +Pi and −Pi condition. Lower panel shows points of root emergence from the side and bottom of basket. **(G)** Percentage of roots emerging from side (outer) and bottom (inner) regions of basket under +Pi and (H) −Pi. Scale bar = 5 cm. ^**^p < 0.01; ^***^p < 0.001; ns not significant as determined by student's *t*-test.

We have recently shown greater biomass accumulation and root hair growth under low Pi of Dular as compared to PB1, which are low Pi tolerant and sensitive genotypes, respectively (Mehra et al., 2015). Here, we analyzed the effects of genotype, treatment and their interaction on growth of 15-days old seedlings under low Pi. Reduction in shoot length was more pronounced in PB1 as compared to Dular under low Pi. However, root length in Dular increased by 33% while PB1 showed an 11% decrease in root length under low Pi (Figure 1A, Table 1). Factorial analysis showed that all the morphological traits were significantly influenced by the low Pi treatment followed by the effect of genotype (Table 1). Interestingly, both genotypes showed a similar increase in root to shoot biomass ratio under Pi deficiency (Figure 1B). Our Two way ANOVA analysis of growth parameters showed that these traits are influenced significantly by the Pi treatment in 15-day-old seedlings (*p* < *0.05*). Intriguingly, Dular has relatively lower soluble Pi content than PB1 in both root and shoot tissues under sufficient Pi. However, under low Pi, Dular showed higher Pi accumulation than PB1 (Table 1).

**Table 1:** **Plant growth and Pi accumulation in Dular and PB1**.

**Genotype**	**Growth condition**	**Root length (cm)**	**Shoot length (cm)**	**Shoot surface Area (cm^2^)**	**Root Pi nmolPi/mg FW**	**Shoot Pi nmolPi/mg FW**
Dular	+Pi	7.03 ± 0.09	24.25 ± 0.39	5.14 ± 0.04	1.65 ± 0.07	9.28 ± 0.37
	−Pi	9.38 ± 0.17	21.9 ± 0.33	4.95 ± 0.03	0.15± 0.01	0.90 ± 0.06
PB1	+Pi	8.31 ± 0.18	21.93 ± 0.30	6.83 ± 0.33	2.27 ± 0.13	11.98 ± 0.41
	−Pi	7.41 ± 0.15	15.36 ± 0.33	3.17 ± 0.10	0.03 ± 0.002	0.73 ± 0.05
**Effects**	**Root length**	**Shoot length**		**Shoot surface area**	**Root Pi**	**Shoot Pi**
Genotype (G)	0.15^*ns*^	1.7E-15^#^		0.8^*ns*^	2.2E-05^#^	0.001^***^
Treatment (T)	0.01^**^	9.6E-20^#^		0.001^***^	9.7E-49^#^	5.1E-39^#^
G × T	4.8E-12^#^	1.3E-05^#^		0.06^*ns*^	2.2E-06^#^	2.5E-06^#^

“+” and “−” indicate 320 μM Pi and 1 μM Pi in nutrient medium, respectively. Values represent mean of at least 20 replicates. ^**^; ^***^; “^#^” indicates highly significant; ns indicate not significant.

### Dular root system architecture seems to be better adapted for low Pi tolerance

RSA analysis revealed greater lateral root length and density in Dular than PB1 which was further increased under low Pi. Interestingly, this increase was higher in Dular as compared to PB1 (Figures 1C–E). Our analysis further showed shallower root growth of PB1 relative to Dular, under both low and sufficient Pi (Figures 1F–H). Interestingly, root spread behavior of both genotypes did not alter significantly under Pi deficiency. However, the total number of roots decreased under low Pi in PB1 (Figures 1F–H).

### Gene expression profiles under Pi deficiency

**Figure 2:**
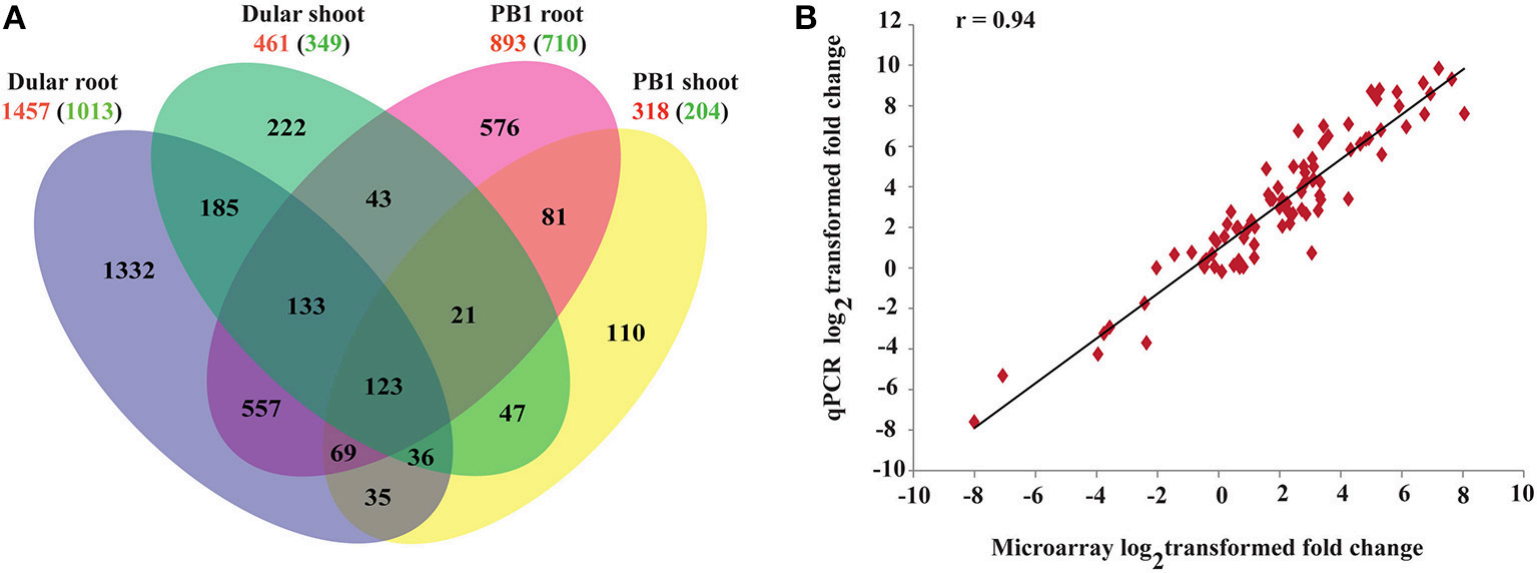
**Gene expression profiles of 15-days-old PB1 and Dular seedlings under Pi deficiency**. **(A)** Venn diagram showing unique and commonly regulated low Pi responsive genes (FC ≥ 2) in PB1 and Dular. Numbers outside and inside the parenthesis indicate up and down-regulated genes, respectively. **(B)** Confirmation of microarray results with qPCR experiments. *r*-value represents the correlation coefficient between log_2_transformed fold changes of microarray and qPCR results.

To understand the molecular basis of differential low Pi tolerance in two genotypes, we performed global transcriptome analysis using microarrays. Three-way ANOVA analysis on microarray data distinguished the low Pi responsive genes from those affected by genotype and tissues independent of Pi levels. Out of total 57,381 probe sets on GeneChip, an overall 39,243 probe sets “significantly expressed' at *p* ≤ 0.05 in either of the tissues, genotypes and Pi treatment. These 39,243 probe sets represented 16,458 unique genes. Of these, expressions of 12,619, 8135, and 13,541 genes were influenced by the main effects of genotype, treatment and tissue, respectively. Whereas, expressions of 4230, 8466, 3379, and 1181 genes were influenced by the effect of interactions of Genotype × Phosphate (G × Pi), Genotype × Tissue (G × T), Tissue × Phosphate (T × Pi) and Genotype × Tissue × Phosphate (G × T × Pi), respectively (Supplementary Figure 1). Out of 8135 low Pi affected genes (Supplementary Table 3), 77% and 84% were also influenced by the variability of genotype and tissue, respectively. Further, 55% of genes were influenced by the two way interaction of G × T while 32% and 33% of Pi responsive genes were influenced by G × Pi and T × Pi interactions, respectively. Only 11% of Pi responsive genes were influenced by the 3-way interactions of G × T × Pi. This analysis revealed high order complexity and intricacy of all three variables. Out of 8135 genes, 1457 genes were up-regulated (−Pi/+Pi) and 1013 were down-regulated (−Pi/+Pi) in Dular roots whereas, in PB1 roots, 893 and 710 genes were significantly (≥2-fold) up and down-regulated, respectively. Shoot tissues, on the contrary, showed a relatively lower number of genes with altered expression (Figure 2A). This indicates more dynamic gene expression in root tissues under Pi deficiency, especially in the low Pi tolerant Dular genotype. Out of 12,619 genes influenced by the genotype factor (Dular/PB1), 4708 and 3908 genes were differentially regulated in root and shoot tissues under Pi deficiency, respectively. Comprehensive analysis of differentially expressed genes showed perturbations in a variety of cellular responses directed toward mitigation of Pi deficiency and other regular plant processes (Supplementary Figure 2). Further, we found many Phosphate Starvation Response (PSR) genes like PHO1, phosphatase, SPX domain containing sequences and others among the differentially expressed genes in both genotypes (Supplementary Figure 3).

Results of the microarray experiments were also successfully validated using qRT-PCR (Figure 2B; Supplementary Table 4) for randomly selected genes with a correlation coefficient >0.94.

### Expression profile of known PSR genes in dular and PB1 under Pi deficiency

**Figure 3:**
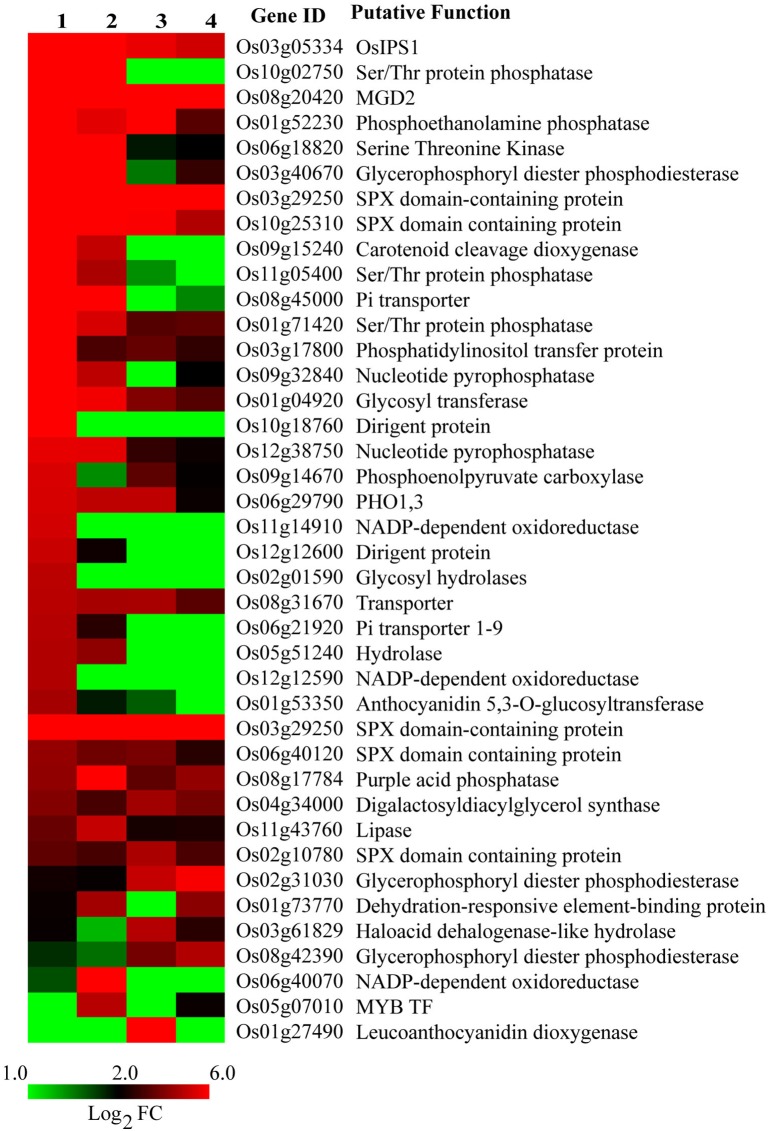
**Expression profile of known PSR genes under Pi deficiency**. Gene list was compiled from published reports. 1, Dular root; 2, PB1 root; 3, Dular shoot; 4, PB1 shoot.

Many PSR genes have been identified and characterized for their roles in improving plant Pi deficiency tolerance. We compiled a list of 40 such common genes by screening the published literature and analyzed their expression behavior in our data (Figure 3). Interestingly, >30 of them were prominently up-regulated in roots, with higher expression levels in Dular than PB1. *OsIPS1* and a ser/thr phosphatase were up-regulated >900-fold in Dular roots. Other Pi responsive genes like SPX domain containing proteins, *PHO* genes, acid phosphatases, phosphoethanolamine phosphatase, and nucleotide pyro-phosphatase were also highly up-regulated in Dular roots as compared to PB1 roots. However, Pi transporter and purple acid phosphatase (Os08g17784) showed greater up-regulation in PB1 roots. We also compiled publically available gene expression data of different rice genotypes under Pi deficiency. Comparison of our data with this list revealed a number of common and many unique PSR genes identified in our study (Supplementary Tables 5, 6).

### Expression analysis of genes involved in internal Pi utilization

#### Induction of transcripts involved in glycolytic bypasses

**Figure 4:**
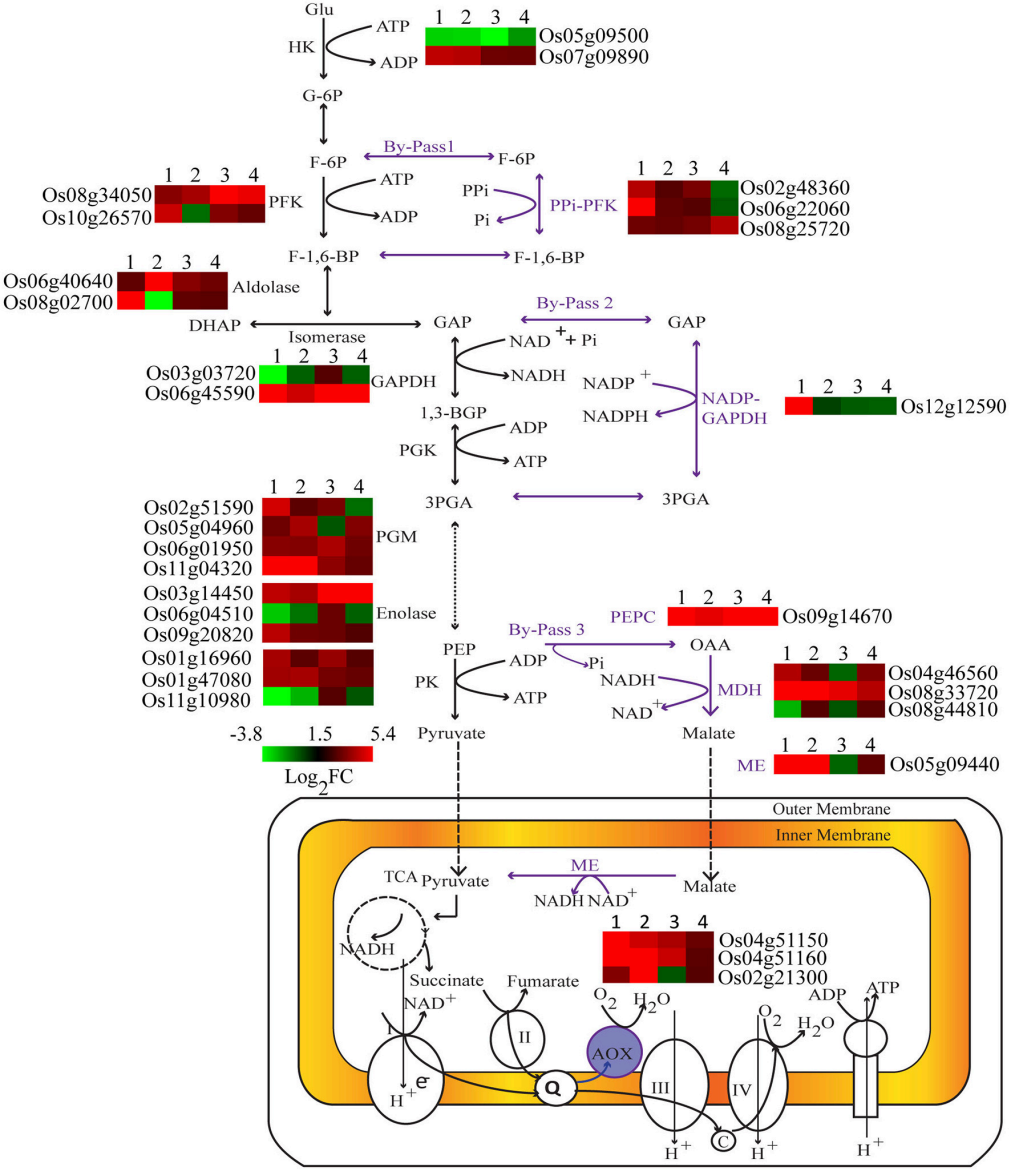
**Differential expression of key genes involved in carbohydrate metabolism under Pi deficiency**. Expression profiles of differentially expressed genes regulating key metabolic reactions are shown adjacent to their enzyme products. 1, Dular root; 2, PB1 root; 3, Dular shoot; 4, PB1 shoot.

Under Pi deprivation, plant cells bypass ATP or Pi dependent biochemical reactions of sugar metabolism using alternate Pi-independent enzymes. Genes involved in three such glycolytic bypasses were altered in both genotypes with higher induction in Dular (Figure 4). The first bypass event is catalyzed by PPi-dependent phosphofructokinase (PPi-PFK), which converts the fructose-6 phosphate to fructose 1, 6-bisphosphate without consumption of ATP. Two PPi-PFK genes, Os02g48360 and Os06g22060, were up-regulated only in Dular roots. However, one such gene (Os08g25720) was also induced in the shoot of PB1. In the second bypass, NADP-GAPDH circumvents the Pi requiring NAD-GAPDH enzyme for the formation of 1, 3-bisphosphoglycerate. The gene (Os12g12590) encoding NADP dependent aldehyde dehydrogenase was specifically up-regulated in Dular roots. Further downstream in glycolysis, one more alternate pathway operates through phosphoenol pyruvate carboxylase (PEPC) and malate dehydrogenase (MDH) to bypass the Pi utilizing pyruvate kinase. In both Dular and PB1, a PEPC encoding gene, Os09g14670, and one MDH encoding gene, Os08g33720 were prominently induced under low Pi treatment. Such adjustment mechanisms were induced in both the genotypes; with a biased overrepresentation in Dular.

#### Dular showed higher expression of lipid remodeling genes

Pi deficiency leads to global membrane lipid remodeling in order to release Pi from membrane phospholipids (Table 2). This involves two major steps, degradation of phospholipids to diacylglycerol and subsequent conversion into galactolipids and sulfolipids. Phospholipid hydrolysis is either mediated by phospholipase C (PLC) in a single step reaction or in two-steps by phospholipase D (PLD) and phosphatidate phosphatase (PAH) (reviewed in Nakamura, 2013). PLC and PLD encoding genes were significantly up-regulated in Dular and PB1 roots and PB1 shoot under low Pi (Table 2). Further, two *PAH* genes (Os11g40080, Os05g38720) were also preferentially up-regulated in Dular, especially in root tissue. In a second step, the major product of phospholipid hydrolysis, diacylglycerol is channeled into biosynthesis of galactolipids, monogalactosyldiacylglycerol (MGDG), and digalactosyldiacylglycerol (DGDG) in plastid membrane (Nakamura, 2013). We found up-regulation of MGDG synthase (Os08g20420) in both genotypes with relatively higher expression in Dular roots. Noticeably, higher up-regulation in Dular shoots was also observed for genes encoding DGDG (Os03g11560, Os04g34000).

**Table 2:** **Expression profile of differentially expressed genes encoding key enzymes in lipid metabolic pathway**.

**Gene ID**	**Functional annotation**	**A. fold change (−Pi/+Pi)**				**B. fold change (Dular/PB1)**			
		**Dular root**	**PB1 root**	**Dular shoot**	**PB1 shoot**	**Root +Pi**	**Root −Pi**	**Shoot +Pi**	**Shoot −Pi**
Os01g19390	ATS	2.94	1.45	1.08	1.02	−1.19	1.70	1.01	−1.04
Os01g22560	ATS	−1.06	−1.01	−7.79	1.56	1.07	1.03	−7.98	135.54
Os05g38350	ATS	5.49	1.77	1.09	1.67	−7.65	−2.47	−3.76	1.25
Os05g38720	PAH	4.37	1.56	1.50	1.36	−1.73	1.61	−1.69	1.16
Os11g40080	PAH	4.01	2.87	3.12	2.20	−1.13	1.23	1.15	1.47
Os03g30130	PLC	4.13	2.63	1.81	2.93	1.82	2.86	1.70	1.44
Os12g37560	PLC	1.56	2.00	1.15	1.11	−1.72	−2.22	−1.26	−1.44
Os06g40170	PLD	3.14	4.02	1.39	2.08	−1.46	−1.87	−2.60	2.43
Os06g40180	PLD	3.47	2.73	1.30	1.37	−1.24	1.03	−5.63	−2.64
Os08g20420	MGD2	627.68	190.77	110.82	123.37	−1.05	3.13	−1.04	16.43
Os03g11560	DGD2	4.20	2.36	3.46	2.03	−1.25	1.43	−1.49	1.35
Os04g34000	DGD2	16.95	8.70	23.15	14.54	−2.80	−1.44	−3.24	5.67
Os05g32140	SQD1	3.51	8.23	2.45	2.89	2.02	−1.16	1.17	2.28
Os01g04920	SQD2	82.66	56.18	16.50	10.19	1.20	1.76	−1.07	3.38
Os07g01030	SQD2	4.40	5.31	2.48	1.81	1.37	1.14	−1.45	2.40
Os01g55780	GDPD	2.31	1.53	1.67	1.28	−1.13	1.33	−1.15	1.08
Os02g31030	GDPD	4.97	4.38	34.50	60.46	1.15	1.30	1.61	1.73
Os03g40670	GDPD	403.33	80.96	2.89	7.23	−6.06	−1.22	1.46	3.06
Os08g42390	GDPD	3.54	2.96	14.35	27.58	2.19	2.62	1.38	1.46

ATS, Acetyl Transferase; PAH, phosphatidate phosphatase; PLC, Phospholipase C; PLD, Phospholipase D; SQD1/2, UDP-sulfoquinovose synthase1/2; GDPD, glycerophosphodiester phosphodiesterases.

Genes involved in sulfolipid biosynthesis, UDP-sulfoquinovose synthase (SQD1) and sulfolipid synthase (SQD2), also showed significant induction in Dular and PB1, especially in roots. SQD2 encoding gene, Os01g04920, was expressed at ≥80-fold in Dular roots and 56-fold in PB1 roots under Pi deficiency. In an alternative pathway, phospholipids hydrolysis is mediated by glycerophosphodiester phosphodiesterases (GDPD). Further analysis revealed four genes encoding GDPD were up-regulated. Of these, Os03g40670 was highly up-regulated in Dular roots (Table 2). An overall fairly high induction of lipid metabolism genes in Dular indicates better cellular Pi homeostasis in this genotype under Pi starvation. To confirm this hypothesis, we analyzed the effect of “Genotype” (G) factor by drawing a comparison of absolute level of transcripts (signal intensities) in Dular with respect to PB1 (i.e., Dular/PB1) under both Pi deficient and sufficient conditions (Table 2). Our analysis confirmed that Dular has higher absolute expression of lipid remodeling genes in shoot and to a lesser extent in roots as well, under Pi deficient conditions when compared to PB1. It is noteworthy from Table 2 that under the sufficient Pi condition Dular has lesser transcripts than PB1 in both root and shoot. Our analysis revealed that expression of lipid remodeling genes more strongly regulated by the genotype than Pi stress. Thus, these genes can contribute to the low Pi tolerance of Dular.

#### Lipid phenotyping revealed greater metabolic flexibility of dular under low Pi

We validated the alterations in transcript abundance of lipid remodeling genes by comprehensive metabolic phenotyping (Supplementary Table 7). Our analysis of relative lipid composition (+Pi/−Pi) revealed decreases in phospholipids such as PC (Phosphatidylcholine), PE (Phosphatidylethanolamine), and PI (Phosphatidylinositol), with a subsequent increase in galactolipids (e.g MGDG, DGDG) under low Pi (Tables 3, 4). Our lipidome analysis also captured the genotypic variability (Dular/PB1) in levels of phospholipids. Under Pi sufficient and deficient conditions, Dular shoots showed higher contents of phospholipids as compared to PB1 shoots (Table 3). On the other hand, PB1 roots and shoots showed a significant decrease in phospholipid content as compared to Dular under Pi deficiency. This indicates faster degradation of phospholipids (i.e., degradation of major proportion of phospholipids) in PB1 under Pi deficiency from its limited reserves. This further implies that Dular has a greater phospholipid reserve to release Pi under low Pi and maintains membrane integrity at the same time. Moreover, there was significant accumulation of phospholipids PC and PE in Dular roots under low Pi. All these evidences reveal greater potential of Dular to cope with low Pi stress than PB1.

**Table 3:** **LC-QTOF analysis of relative concentrations of phospholipids under Pi deficiency**.

**m/z**	***t*_*R*_**	**Annotation**	**A. peak ratios (−Pi/+Pi)**				**B. peak ratios (Dular/PB1)**			
			**Dular root**	**PB1 root**	**Dular shoot**	**PB1 shoot**	**Root +Pi**	**Root −Pi**	**Shoot +Pi**	**Shoot −Pi**
734.57	5.44	PC (16:0/16:0) [M+H]+	0.08	0.04	0.1	0.05	1.12	2.15	2.29	4.26
732.55_754.54	4.92_4.79	PC (32:1) [M+H]+_PC (32:1) [M+Na]+	0.39	0.06	0.16	0.13	0.41	2.84	2.24	2.83
752.52_730.54	4.43_4.44	PC (32:2) [M+Na]+_PC (32:2) [M+H]+	0.27	0.09	0.12	0.04	0.62	1.81	2.88	10.13
744.55_766.54	4.73_4.77	PC (33:2) [M+H]+_PC (33:2) [M+Na]+	0.44	0.07	0.23	0.06	0.44	2.71	1.54	6.18
742.54	4.34	PC (33:3)	0.5	0.07	0.24	0.06	0.36	2.49	2.95	11.68
760.58	5.76	PC (34:1) A [M+H]+	0.32	0.14	0.08	0.32	0.5	1.15	2.99	0.79
760.58_782.57	5.52_5.52	PC (34:1) B [M+H]+_PC (34:1) B [M+Na]+	0.3	0.1	0.17	0.05	0.46	1.38	2.66	9.08
758.57	5.19	PC (34:2) A [M+H]+	0.3	0.07	0.22	0.08	0.68	2.71	2.9	7.52
780.55_758.57	5.03_5.03	PC (34:2) B [M+Na]+_PC (34:2) B [M+H]+	0.41	0.18	0.32	0.09	0.72	1.69	1.82	6.72
756.56	4.95	PC (34:3) A [M+H]+	0.47	0.41	0.35	0.25	0.25	0.28	3.15	4.53
756.55_778.54	4.61_4.61	PC (34:3) B [M+H]+_PC (34:3) B [M+Na]+	0.45	0.17	0.45	0.1	0.61	1.64	2.77	13.03
754.54	4.26	PC (34:4)	0.42	0.1	0.15	0.15	0.54	2.34	5.37	5.29
772.58	5.05	PC (35:2) A [M+H]+	0.48	2.13	1.8	0.39	2.33	0.52	0.6	2.81
772.59	5.39	PC (35:2) B [M+H]+	0.31	0.32	0.22	0.3	0.71	0.7	2.42	1.78
770.57	4.90	PC (35:3) B [M+H]+	0.39	0.12	0.31	0.52	0.66	2.09	4.26	2.55
788.62_810.60	6.17_6.17	PC (36:1) [M+H]+_PC (38:4) B [M+H]+	0.14	0.15	0.27	0.1	1.02	0.98	1.72	4.53
786.60_808.58	5.63_5.64	PC (36:2) [M+H]+_PC (36:2) [M+Na]+	0.18	0.14	0.32	0.09	0.59	0.72	1.74	6.15
784.59	5.13	PC (36:3) A [M+H]+	0.5	0.15	0.17	0.05	0.42	1.45	2.44	7.83
804.55_782.57	4.65_4.64	PC (36:4) A [M+H]+_PC A (36:4) [M+Na]+	0.52	0.19	0.33	0.11	0.57	1.59	1.6	5
804.55	5.04	PC (36:4) B [M+Na]+	0.21	0.41	0.83	0.47	1.38	0.69	1.07	1.92
780.55	4.29	PC (36:5) [M+H]+	0.46	0.11	0.39	0.12	0.37	1.49	2.61	8.76
778.54	3.91	PC (36:6) [M+H]+ A	0.39	0.08	0.35	0.12	0.23	1.14	6.78	19.93
800.62	6.04	PC (37:2) [M+H]+	0.11	0.21	0.67	0.88	1.25	0.66	1.92	1.46
777.55_794.58	4.73_4.72	PC (37:5) [M+H]+_PC (37:5) [M+NH4]+	0.2	0.14	1.61	0.61	0.43	0.6	0.62	1.63
808.58_808.58	4.78_5.03	PC (38:5) A [M+H]+_PC (38:5) A [M+H]+	0.44	0.49	0.23	0.25	1.03	0.91	2.22	2.05
806.57	4.69	PC (38:6) A [M+H]+	0.57	0.38	0.34	0.22	0.91	1.36	1.68	2.58
806.57	5.10	PC (38:6) B [M+H]+	0.52	0.16	0.15	0.09	0.42	1.37	2.86	5.02
742.58	5.15	PC (p-34:2) or PC (o-34:3) [M+H]+	0.7	0.99	0.44	0.67	1.34	0.95	1.46	0.96
798.64	6.44	PC (p-38:2) or PC (o-38:3) [M+H]+	0.48	0.63	0.52	0.79	1.02	0.77	1.35	0.89
794.60	5.78	PC (p-38:4) B or PC (o-38:5) B [M+H]+	0.34	0.26	0.45	0.7	0.63	0.82	1.73	1.13
716.52_738.51	5.18_5.19	PE (34:2) [M+H]+_PE (34:2) [M+Na]+	0.14	0.11	0.08	0.03	0.89	1.18	2.68	8.04
740.53	4.80	PE (36:4) [M+H]+	0.21	0.1	0.16	0.05	0.48	1	2.36	7.13
714.51	5.21	PE (34:2) [M−H]− B	0.16	0.12	0.1	0.03	0.83	1.11	2.6	7.87
740.52	5.29	PE (36:3) [M−H]− B	0.4	0.21	0.5	0.35	0.59	1.1	2.49	3.56
818.59	6.11	PE (38:1) [M+HCOO]− A	0.76	1.65	0.49	0.48	0.91	0.42	1.39	1.45
747.52	5.04	PG 34:1 [M−H]−	0.04	0.03	0.23	0.15	0.52	0.57	1.94	2.97
745.50	4.62	PG 34:2 [M−H]−	0.11	0.06	0.07	0.13	0.53	0.87	2.58	1.41
819.53	4.32	PG 40:7 [M−H]−	0.14	0.13	1.01	0.51	0.46	0.48	0.98	1.94
835.53	4.92	PI (34:1) [M−H]−	0.31	0.21	0.46	0.44	0.66	0.97	0.88	0.93
901.51 and 833.52	4.48 and 4.45	PI (34:2) [M+NaHCO2]− & PI (34:2) [M−H]−	0.21	0.23	0.15	0.13	0.77	0.71	0.96	1.12
857.52	4.14	PI 36:4 [M−H]−	0.18	0.28	0.19	0.26	0.83	0.52	0.64	0.47

PC, PhosPC: photidylcholine; PE, Phosphotidylehanolamine; PG, Phosphotidylglycerol; PI, Phosphotidylinositol.

**Table 4:** **LC-QTOF analysis of relative concentrations of galactolipids under Pi deficiency**.

**m/z**	***t_R_***	**Annotation**	**A. peak ratios (-Pi/+Pi)**				**B. peak ratios (Dular/PB1)**				**C. peak ratios (Shoot/Root)**			
			**Dular Root**	**PB1 Root**	**Dular Shoot**	**PB1 Shoot**	**Root +Pi**	**Root −Pi**	**Shoot +Pi**	**Shoot −Pi**	**Dular +Pi**	**PB1 +Pi**	**Dular −Pi**	**PB1 −Pi**
941.6176_936.6609	5.538_5.51	DGDG 34:1 [M+Na]+ DGDG 34:1 [M+NH4]+	1.54	1.71	1.19	1.32	0.60	0.54	0.51	0.46	0.94	1.10	0.73	0.85
962.6771	5.69	DGDG 36:2 [M+NH4]+	2.08	1.81	3.01	2.25	0.70	0.80	0.51	0.69	2.21	3.02	3.20	3.74
965.6163_960.6603	5.206_5.18	DGDG 36:3 [M+Na]+ DGDG 36:3 [M+NH4]+	3.26	2.40	1.96	1.02	0.67	0.91	0.61	1.18	6.45	7.09	3.88	3.01
961.5859_956.6313	4.267_4.27	DGDG 36:5 [M+Na]+ DGDG 36:5 [M+NH4]+	0.90	0.56	2.26	2.08	0.41	0.65	0.63	0.69	5.42	3.46	13.68	12.82
954.6155	3.92	DGDG 36:6 [M+NH4]+	0.16	0.15	1.17	0.84	0.36	0.38	0.91	1.26	28.33	11.33	209.7	63.02
779.5652	6.03	MGDG 34:1 [M+Na]+	0.68	2.63	1.01	0.37	0.78	0.20	0.61	1.69	0.86	1.10	1.29	0.15
777.5495	5.54	MGDG 34:2 [M+Na]+	0.61	2.68	1.73	0.74	1.25	0.29	0.62	1.47	0.49	0.99	1.39	0.27
773.5189	4.59	MGDG 34:4 [M+Na]+	0.08	0.03	0.96	0.16	0.44	1.01	0.44	2.57	1.29	1.30	16.20	6.38
803.5664_798.6027	5.681_5.54	MGDG 36:3 [M+Na]+ MGDG 36:3 [M+NH4]+	0.78	1.02	1.43	0.37	0.39	0.30	0.70	2.71	3.00	1.69	5.47	0.61
799.5335	4.70	MGDG 36:5 [M+Na]+	0.28	0.33	1.37	0.74	0.62	0.53	0.70	1.30	2.27	2.02	10.91	4.49
797.5183_792.5621	4.301_4.31	MGDG 36:6 [M+Na]+ MGDG 36:6 [M+NH4]+	0.10	0.11	1.02	0.47	0.48	0.44	0.96	2.11	6.76	3.34	71.60	14.87
770.5749	5.11	MGDG 34:3 [M+NH4]+	0.38	0.47	1.75	0.39	0.54	0.43	0.61	2.75	0.94	1.10	0.73	0.85

MGDG, Monogalactosyldiacylglycerol; DGDG, Digalactosyldiacylglycerol.

Interestingly, low Pi-tolerant Dular also showed increased accumulation of galactolipids, DGDG in both roots and shoots under low Pi as compared to PB1. While, increased accumulation of MGDG was found in Dular shoots under low Pi (−Pi/+Pi) (Table 4). Genotypic analysis (Dular/PB1) revealed increased accumulation of MGDG and DGDG in Dular shoots as compared to PB1 under Pi deficiency (Table 4). Noticeably, these observations are in high concordance with transcriptome results of shoot tissue in both the genotypes. However, PB1 root tissue had a higher accumulation of galactolipids than Dular under Pi deficiency (Table 4). To assess the impact of this observation, we further analyzed the lipid data in terms of third factor, “Tissue” (Shoot/Root; Table 4). Interestingly, we found higher accumulation of galactolipids in shoots than roots. This differential accumulation accounts to the fact that photosynthetic leaves contain chloroplasts with larger surface area as compared to roots with rudimentary plastids. Galactolipids form the bulk of these shoot thylakoid membranes as they play important role in light reactions (Dormann, 2007). Thus, higher accumulation of galactolipids in Dular shoots might outpace the advantage of having higher accumulation of galactolipids in PB1 roots under Pi deficiency.

### Expression analysis of genes involved in Pi acquisition

#### Induction of RSA, cell wall loosening, and biosynthesis genes

Pi deficiency generally alters root architecture for increasing soil foraging capacity. Therefore, we compared our data with available transcriptome data of rice roots in different developmental stages and tissues (Takehisa et al., 2012). Our analysis revealed upregulation of 64 genes involved in lateral root development in Dular roots, but upregulation of only 33 genes in PB1 (Supplementary Table 8). Furthermore, cell wall loosening and biosynthesis are essential processes for modulating RSA. A comparison of our data with the Cell Wall Navigator Database (Girke et al., 2004) revealed higher numbers of up-regulated root growth and cell wall biosynthesis genes in Dular roots (66) as compared to PB1 roots (34). These genes include expansins, glycosyl transferases, glycosyl hydrolases and cellulose synthase (Supplementary Table 9 and Supplementary Figure 4). A comparative analysis with rice GT genes database (Cao et al., 2008) showed 109 differentially expressed genes in both the genotypes (Supplementary Table 10). Interestingly, Dular roots showed a higher number of up-regulated GT genes (82) than PB1 roots (37). Five of the xyloglucan endotransglucosylases (Os04g51460, Os06g48200, Os07g34580, Os03g02610, and Os03g63760) were also found significantly upregulated in Dular roots under Pi deficiency. This again showed the general tendency of higher up-regulation of transcripts related to root growth and cell wall loosening in Dular root as compared to PB1 roots.

#### Increased expression of acid phosphatases and organic acids genes in dular

**Figure 5:**
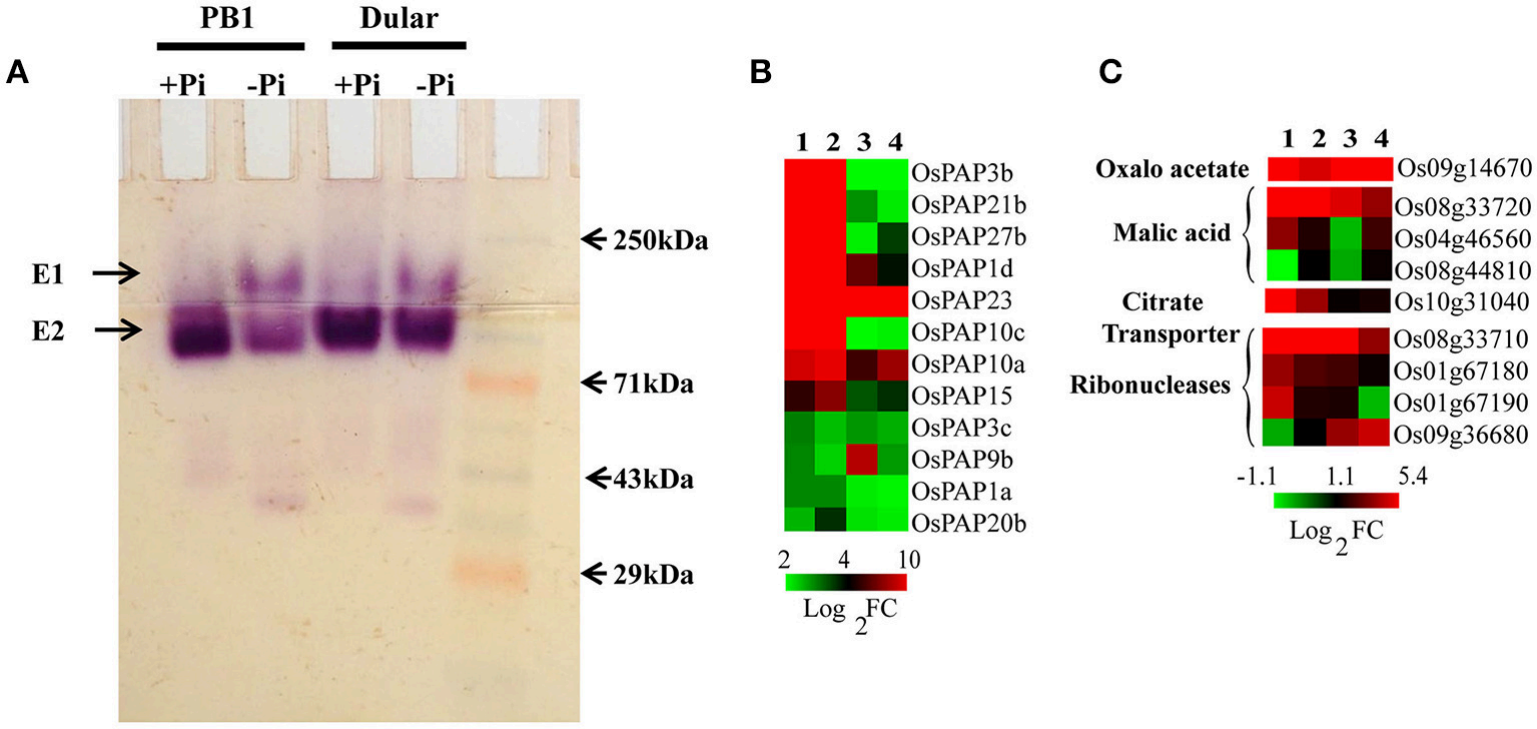
**Effect of Pi deficiency on APases and genes involved in organic acids metabolism**. **(A)** In-gel APase activity of proteins extracted from PB1 and Dular roots under +Pi and −Pi conditions. **(B)** Expression profile of APases showing higher expression of APases in Dular and PB1 under +Pi and −Pi. **(C)** Differential expression of genes involved in organic acid metabolism in Dular and PB1 under Pi deficiency. 1, Dular root; 2, PB1 root; 3, Dular shoot; 4, PB1 shoot.

Phosphatases, ribonucleases and organic acids release the Pi from organic/inorganic compounds in the rhizosphere under its deficiency. We found 266 various types of phosphatases and hydrolases differentially expressed in Dular and PB1, out of which, 128 and 95 genes were up-regulated in Dular and PB1 roots, respectively (Supplementary Table 11). Interestingly, up-regulation of ser/thr phosphatase (Os10g02750) and phosphocholine phosphatase (Os01g52230) genes was as high as 909-and 552-fold in Dular roots in comparison to 318-and 47-fold in PB1 roots, respectively. In-gel APase assay also revealed the higher activity of APases under Pi deficiency in Dular roots as compared to PB1 (Figure 5A). An earlier reported Pi inducible E1 isoform of APase, OsPAP10, was found to be stained with equal intensity under Pi deficiency. The gene for the same isoform also showed almost equal upregulation of 9.16 and 9.62 in Dular and PB1 roots, respectively.

We also found prominent upregulation of a PEPC encoding gene (Os09g14670) in Dular roots and shoots (42-and 11-fold) as compared to PB1 roots and shoots (2.7-and 4.2-fold) which catalyze the synthesis of oxaloacetate and release Pi as a by-product (Supplementary Table 11 and Figure 5B). Additionally, genes involved in malic acid metabolism, (MDH Os04g46560, Os08g33720) were also up-regulated in roots of both genotypes. Moreover, genes for ribonucleases (RNases) (Os08g33710, Os01g67180, Os01g67190) and a citrate transporter (Os10g31040) were also up-regulated more prominently in Dular roots. This comparative study of phosphatases, RNases, and organic acids encoding genes revealed a potential ability to solubilize soil-bound Pi by Dular under Pi deficiency (Supplementary Table 11 and Figure 5).

#### Higher induction of Pi transporter genes in PB1

We observed that 104 transporter encoding genes were upregulated in Dular roots while only 52 were in PB1 under Pi stress (Supplementary Table 12). Out of 26 potential Pi transporters in rice, 5 Pi transporters, including the high affinity Pi transporter *OsPT6*, were induced (Supplementary Figure 5). Interestingly, high affinity Pi transporters like *OsPT6* (Os08g45000) showed higher upregulation in PB1 roots as compared to Dular. Analysis of absolute transcript expression (Dular/PB1) revealed a higher expression (3.6) of *OsPT6* in PB1 roots under Pi deficiency as compared to Dular. Notably, our data also reflect the transcript induction of the *OsPHO1-3* (Os06g29790) gene involved in Pi loading into xylem. Transcripts of four SPX domain containing proteins (Os03g29250, Os10g25310, Os06g40120, and Os02g10780) were also up-regulated in both Dular and PB1 roots.

### Identification of SNPs and indels between dular and PB1 in differentially expressed PSR genes

We also identified SNPs and InDels presents between Dular and PB1 in 8135 differentially regulated genes under low Pi conditions using available genome sequence (Mehra et al., 2015; Supplementary Table 13). 75244 genic SNPs and 16400 genic InDels were identified between Dular and PB1; however, 17739 coding SNPs and 1123 coding InDels were discovered. 5′ UTRs and 3′ UTRs of Dular and PB1 were also analyzed for SNPs and InDels which yielded 3978, 9048 SNPs and 1772 and 2455 InDels, respectively. Functional classification of these SNPs and InDels revealed 10 startloss and 18 stoploss SNPs between Dular and PB1. Moreover, 69 non-sense and 7337 missense as well as 7805 silent SNPs were identified. Further, 217 large effect frameshift InDels between Dular and PB1 were also observed. Additionally, we also analyzed the SNPs and InDels present in 2kb upstream promoter regions of key PSR genes (Supplementary Table 14). Interestingly, numerous SNPs and InDels were found in promoter regions of lipid remobilizing genes (*SQD2, GDPD, ATS, PAH, MGD2, DGD2, PLD*), a high affinity phosphate transporter (*OsPT6*), SPX domain containing proteins, various organic acid genes (lactate/malate dehydrogenase, *PEPC*) and purple acid phosphatases between Dular and PB1 (Supplementary Table 14).

## Discussion

We investigated the potential low Pi tolerance mechanisms using comparative morphophysiological, transcriptomics, and lipidomics approaches in a low Pi sensitive modern rice genotype (PB1) and a tolerant genotype, Dular. Pusa Basmati-1 (PB1) is the first high yielding semi-dwarf Basmati variety which yields ~50–52 q/ha and shows improved growth parameters with high nutrient supply (Sharma et al., 2012). On the other hand, Dular is a traditional genotype with very low yield potential of about 7–22 q/ha and has been shown to be one of the most tolerant rice genotypes for Pi deficiency in field conditions (Wissuwa and Ae, 2001; Chin et al., 2010). Low Pi tolerance can be achieved through better Pi acquisition and efficient cellular Pi utilization (Wang et al., 2010; Rose et al., 2011). Higher “Pi acquisition efficiency” corroborates to enhanced Pi uptake, root architectural modifications and solubilisation of bound Pi in soil whereas “Pi utilization efficiency” refers to efficient remobilization of cellular Pi. Generally, modern genotypes are believed to be highly “Pi responsive” due to higher Pi uptake, whereas, traditional genotypes like Dular are “Pi efficient” (Gerloff, 1977; Wissuwa and Ae, 2001). However, insufficient molecular evidences exist to support this notion. Therefore, we employed transcriptome and lipidome profiling of both contrasting genotypes to reveal the low Pi tolerance mechanisms of a Pi efficient genotype.

Three way ANOVA analysis of microarray data revealed that most of the transcripts were influenced by tissue and genotype. This suggests that most of the Pi responsive genes were expressed at different background levels in selected genotype and tissue types. We found two- and five-fold higher numbers of differentially expressed transcripts affected by the genotype factor as compared to the Pi treatment factor in Dular root and shoot tissues, respectively. Thus, there exists a significant genetic variability between the selected genotypes which provides a novel aspect to unravel the low Pi tolerance mechanisms. Our integrated transcriptome, lipidome, and morphological analysis revealed that Dular showed multifaceted tolerance mechanisms which involve RSA modulation and lipid remobilization. However, earlier studies conducted by Pariasca-Tanaka et al. (2009) had shown RSA modulation as the only mechanism for low Pi tolerance of NIL6-4 (introgressed with *Pup1* QTL from low Pi tolerant *aus* genotype, Kasalath). This was probably due to less genetic variability between NIL6-4 and low Pi sensitive recurrent parent, Nipponbare. Intriguingly, both PB1 and Dular carry the *Pup1* candidate gene *Pstol1* (data not shown). Therefore, present study becomes more rational to look into the other low Pi tolerance mechanisms. In another study, Oono et al. (2013) found that low Pi induced genes are more up-regulated in tolerant *aus* genotypes, Kasalath than sensitive genotypes. Our study also confirms the same hypothesis in another *aus* genotype Dular and proved the importance of transcriptional regulation for low Pi adaptation. Furthermore, similar to earlier observation in Kasalath (Oono et al., 2013), Dular also showed reduced inorganic P content which reflects on potential efficient P utilization.

RSA modulations like increased root hairs, lateral roots and higher turnover of roots are important traits involved in adaptation to Pi deficiency (Lynch, 2011; Gamuyao et al., 2012; Pandey et al., 2013). Sensing of Pi deficiency at the root tip activates the downstream Pi responsive genes associated with RSA modulation (Svistoonoff et al., 2007). Our transcriptome data also showed higher induction of RSA genes (glycosylases, expansins, XTHs) in Dular roots in comparison to PB1 under Pi deficiency. Phenotypic analysis of Dular and PB1 genotypes also depicted apparent differences in the root architecture of both genotypes. In an earlier study, we found increased lateral roots and root hair growth in Dular under low Pi as compared to PB1 (Figure 1C, Mehra et al., 2015). Both traits bestow enhanced Pi acquisition by increasing the absorptive surface area under Pi limiting condition. Additionally, Dular roots showed a significant increase in root length under low Pi. This quantitative trait has been linked with Pi deficiency tolerance in some rice genotypes (Shimizu et al., 2004). Increased root length is associated with longer and more branched roots per unit of root dry matter (Hill et al., 2006). Root elongation enhances the porosity and oxygen release capacity of plants which leads to iron oxidation and release of protons. This causes an increase in rhizosphere acidity that helps in solubilisation of soil Pi compounds (Kirk and Du, 1997). Therefore, increase in root length helps in surviving in Pi poor soil. Our analysis of root spread showed that PB1 possesses shallow root system which suggests that it has been selected on nutrient rich soils for higher Pi acquisition attributing to its higher yield. Since, PB1 is a drought sensitive genotype (Mutum et al., 2013) shallow root system may contribute to its high drought sensitivity. On the other hand, we observed a steeper-angled root system in Dular which also likely contributes to its drought avoidance mechanism as Dular is a traditional upland genotype (Henry et al., 2011). Moreover, upland systems are co-limited for water and Pi together. Therefore, Dular has been co-selected for both the traits i.e., low Pi tolerance and drought avoidance. Therefore, this traditional “Pi-efficient” genotype can also be further exploited to target the dual problem of drought and low Pi tolerance. We didn't find significant change in root spread for either genotype caused by low Pi. This indicates that root spread behavior might be governed by genotype rather than low Pi stress. Interestingly, we also observed greater cortical aerenchyma formation in Dular than PB1 root under Pi deficiency (Supplementary Figure 6). This adaptive trait allows reallocation of resources toward formation of new roots with minimum additional metabolic cost (Lynch, 2011; Postma and Lynch, 2013). Noticeably, under Pi deficiency, Dular roots also showed higher induction of XTHs (Xyloglucan endotransglucosylase) genes known to be involved in aerenchyma and root hair formation (Vissenberg et al., 2001). It is noteworthy that NIL6-4 with introgressed *Pup1* locus didn't show such advantage in root hair and aerenchyma formation despite induction of XTHs. This implies that these complex traits involve several molecular regulators influenced by environment and genotype. The integration of these root traits may lead to synergistic interactions that substantially increase P uptake (York et al., 2013).

Metabolic plasticity plays an important role in conserving cellular Pi (Plaxton and Tran, 2011) and thereby enhances Pi utilization efficiency. In our data, many key genes involved in glycolytic bypasses were significantly up-regulated in Dular thus efficiently mitigating the demand of Pi under its deficiency. Cellular Pi pools can also be conserved by replacing membrane phospholipids with galactolipids and sulfolipids (Nakamura, 2013; Okazaki et al., 2013). A set of membrane lipid remodeling genes, including like PLC, PLD, and GDPD, were found to be induced in Dular at relatively higher levels than PB1 under low Pi. Further, low Pi induction of lipid remodeling genes and accumulation of corresponding metabolic lipid bio-markers (MGDG and DGDG) indicate that lipid remobilization is also one of adaptive strategies for low Pi tolerance in the traditional genotype. Analysis of phospholipid composition showed higher degradation of phospholipids in PB1 under low Pi. However, the total amount of phospholipids degraded is more in Dular (analogy model, Supplementary Figure 7). Genotypic comparison of Dular and PB1 revealed a greater pool of phospholipids in Dular shoots under both low and high Pi as compared to PB1. This indicates Dular has greater room to maneuvre its phospholipid reserve under low Pi stress. All these results together indicate the higher metabolic adjustments in Dular relative to PB1 which allow it to withstand low Pi stress. However, a matter of future research will be whether high phospholipid content and its remobilization is a general low Pi tolerance strategy among traditional rice genotypes. Earlier reports also suggest lipid remobilization is one of the important low Pi tolerance mechanisms in other crops like kidney bean, oat, and sesame (Gniazdowska et al., 1999; Andersson et al., 2003, 2005; Shimojima et al., 2013).

Moreover, SNPs and InDels identified in the promoter regions between PB1 and Dular can modulate the differential expression of these PSR genes between these two genotypes. This differential expression due to non-sense and other large effect SNPs of Dular and PB1 can provide the tolerance and sensitive attributes to these genotypes. Further characterization of these large effect variants will allow the detailed dissection of the molecular mechanism of low P tolerance of Dular and other traditional cultivars. However, a large population will be needed to explore these SNPs to achieve the necessary genotypic and phenotypic variation.

In our data, we also found induction of various transporters in Dular, including the glucose six phosphate transporter, glycerol three phosphate transporters, and phospholipid transporters, which are involved in cellular Pi remobilization. This provides additional evidence of better internal Pi utilization in Dular. Pi transporters (both low and high-affinity) mediate uptake from soil and distribution of Pi in plants against concentration gradients (Ai et al., 2009; Li et al., 2015). However, induction of the high-affinity Pi transporter was observed as a response rather than a tolerance mechanism under low Pi (Pariasca-Tanaka et al., 2009; Oono et al., 2013). High induction of *OsPT6* in PB1 roots might reflect its greater Pi demand from exogenous sources. On the contrary, Dular's higher Pi use efficiency and low Pi demand is potentially addressed by better cellular Pi mobilization and efficient Pi acquisition. This may explain the relatively lower induction of high affinity Pi transporters in Dular.

In conclusion, we have shown that the traditional genotype Dular might employ both better internal Pi utilization and acquisition strategies for low Pi tolerance as revealed by transcriptome, lipidome and RSA phenotyping. Traditional upland genotypes like Dular can serve as a novel genetic sources for improving low Pi tolerance of modern Pi responsive elite cultivars.

## Author note

Microarray data submitted in Gene Expression Omnibus (GEO) database, www.ncbi.nih.gov/geo (accession no. GSE74795).

## Author contributions

PM and BP conducted the experiments and contributed in writing the manuscript. JG conceived the idea, designed the project, analyzed data, and wrote the manuscript with the help from co-authors.

### Conflict of interest statement

The authors declare that the research was conducted in the absence of any commercial or financial relationships that could be construed as a potential conflict of interest.
