# Supplementary material for: Comparative Morphophysiological Analyses and Molecular Profiling Reveal Pi-Efficient Strategies of a Traditional Rice Genotype
Source: Front Plant Sci. 2016 Jan 5;6:1184. doi: 10.3389/fpls.2015.01184 (PMC4700128; doi:10.3389/fpls.2015.01184)
Supplement: Supplementary file 2 [file Image1.PDF]

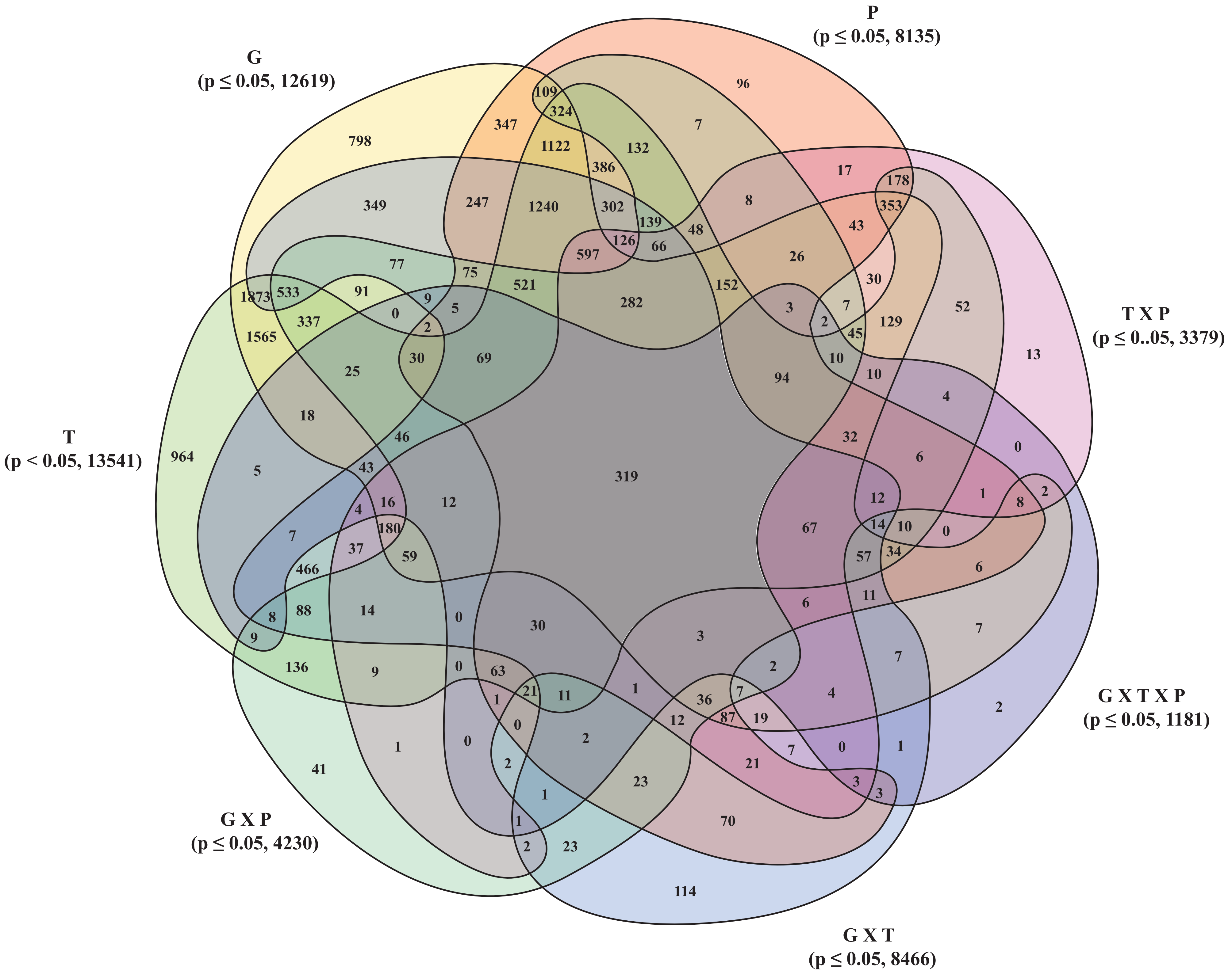

Supplementary Fig. S1. 7-way venn diagram showing unique and overlapping genes significantly expressing (p value ≤ 0.05) under all main and interactions effects of Genotype, G; Tissue, T and Phosphate, Pi.

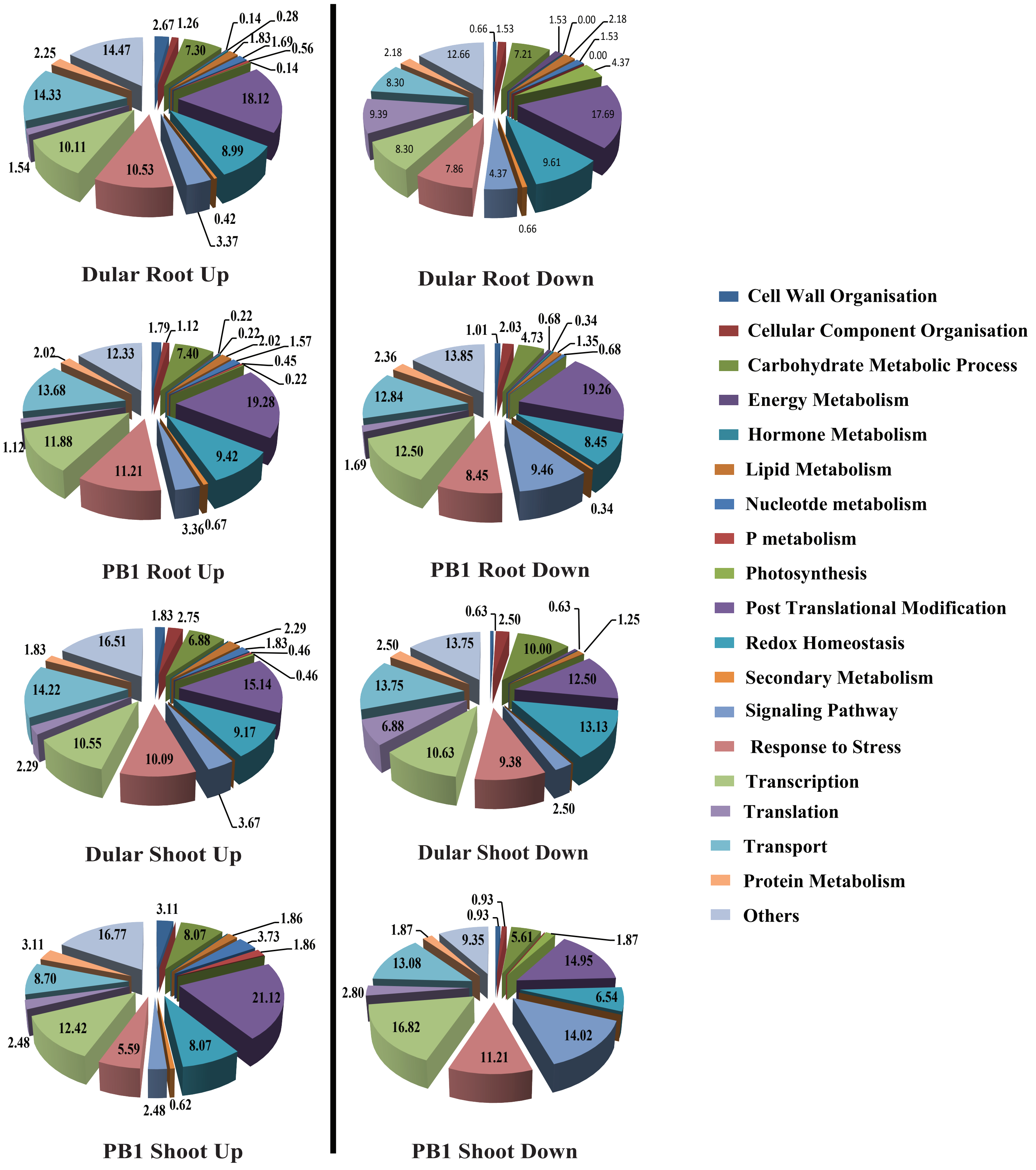

Supplementary Fig. S2. Functional categorisation of genes, up and downregulated in root and shoot tissues at *p* value ≤ 0.05. Functional categories were made according to GO terms assigned in Rice Oligonucleotide Array Database.

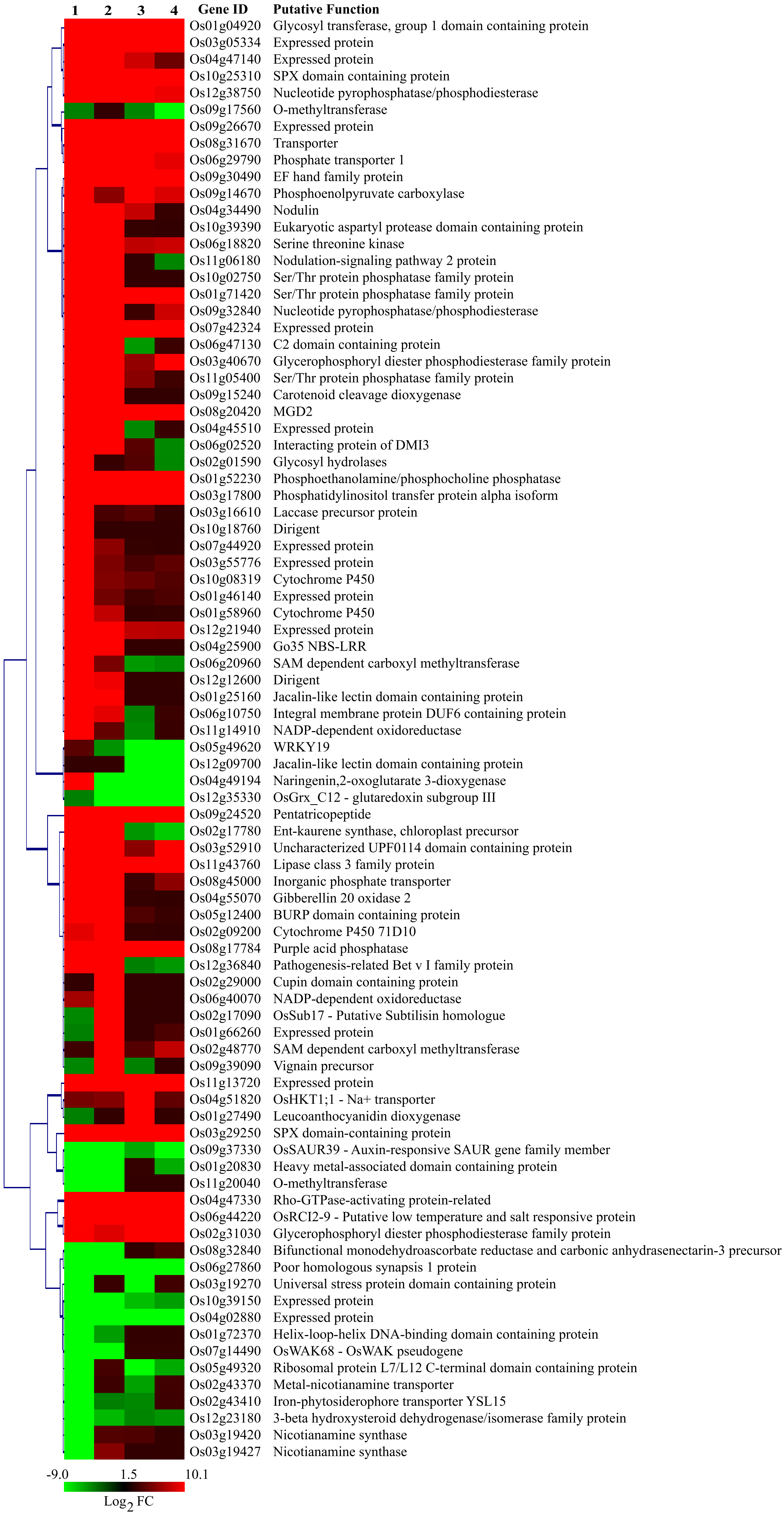

Supplementary Fig. S3. Expression profile of highly induced genes under Pi deficiency. Genes showing ≥ 30 FC in either of the tissues (root or shoot) or genotypes (Dular and PB1) under Pi deficiency were considered. 1, Dular root; 2, PB1 root; 3, Dular shoot; 4, PB1 shoot.

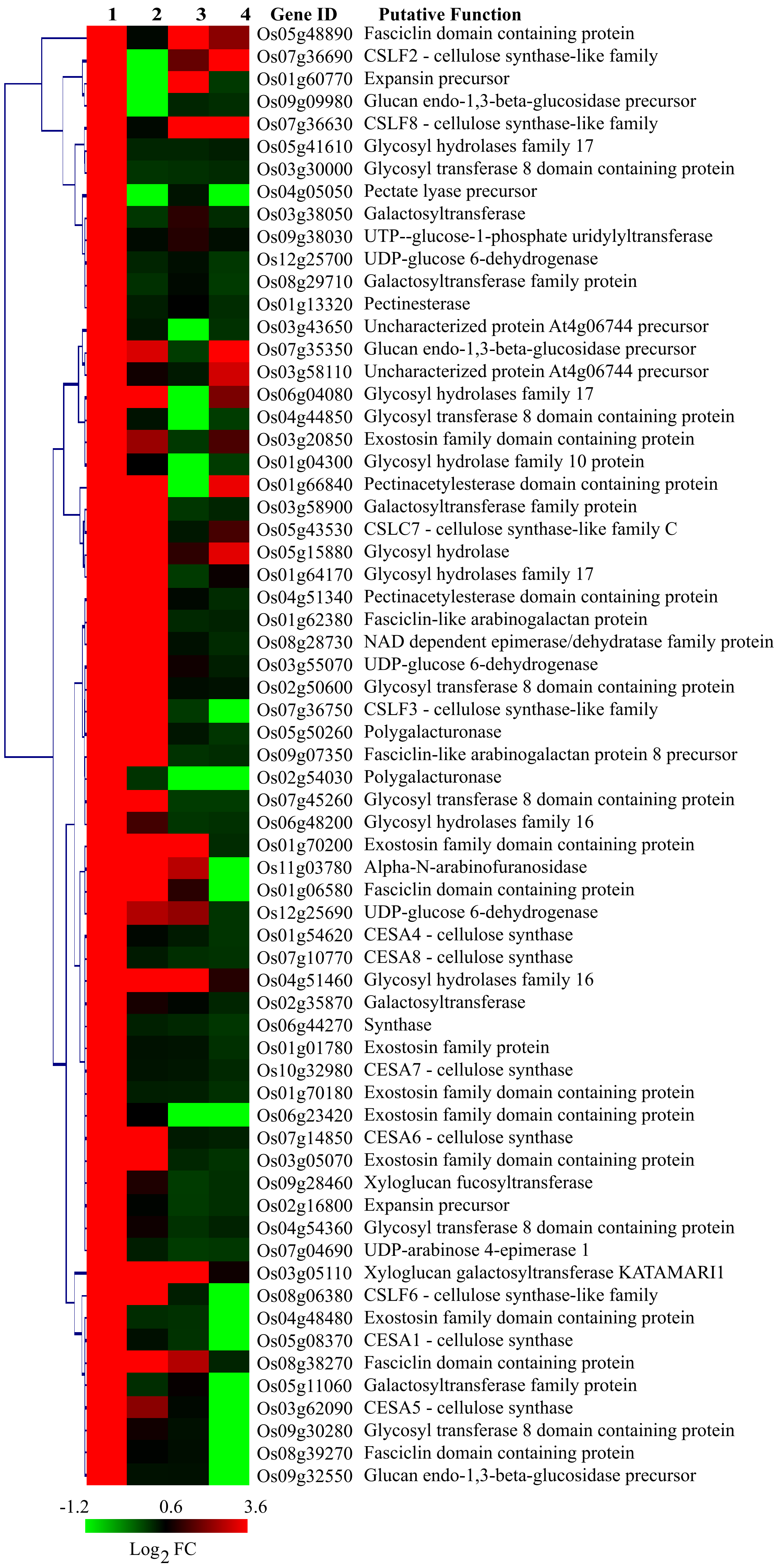

Supplementary Fig. S4. Effect of Pi deficiency on expression profiles of root system architecture and cell wall biosynthesis genes. Potential genes involved in RSA and cell wall modulation were identified from our data using information from previously published reports and cell wall navigator database. 1, Dular root; 2, PB1 root; 3, Dular shoot; 4, PB1 shoot.

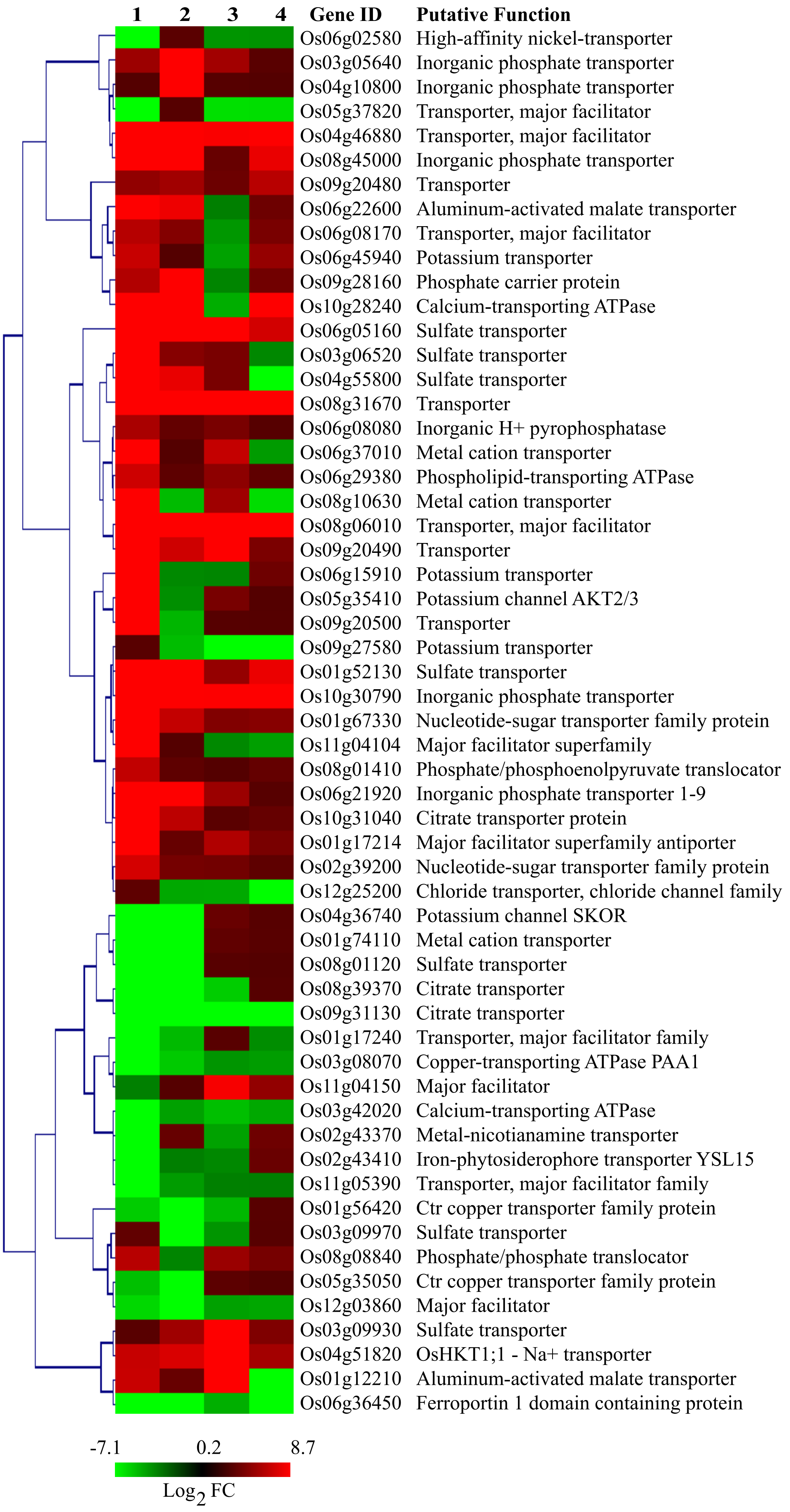

Supplementary Fig. S5. Expression profiles of membrane transporter encoding genes showing significant expression in Dular and PB1 root and shoot tissues. 1, Dular root; 2, PB1 root; 3, Dular shoot; 4, PB1 shoot.

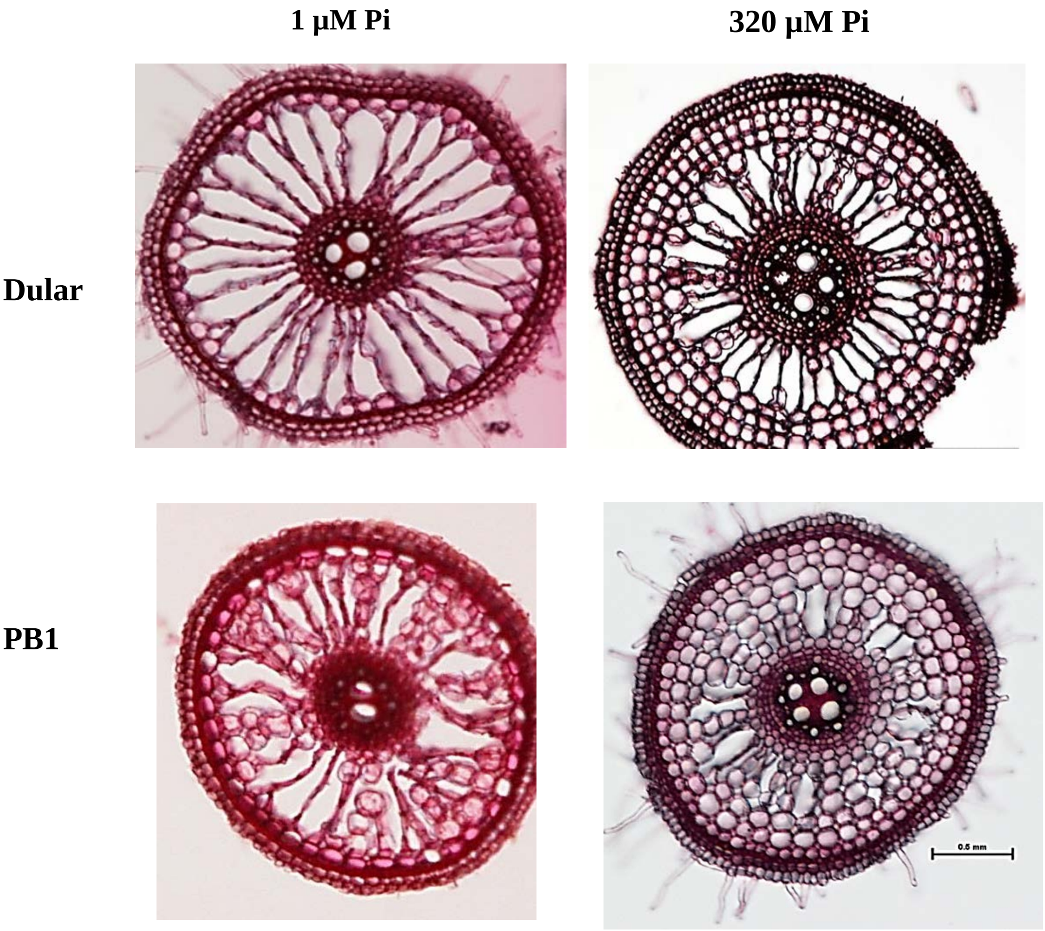

Supplementary Fig. S6. Effect of Pi deficiency on aerenchyma formation in root tissues of 15-days-old seedlings.

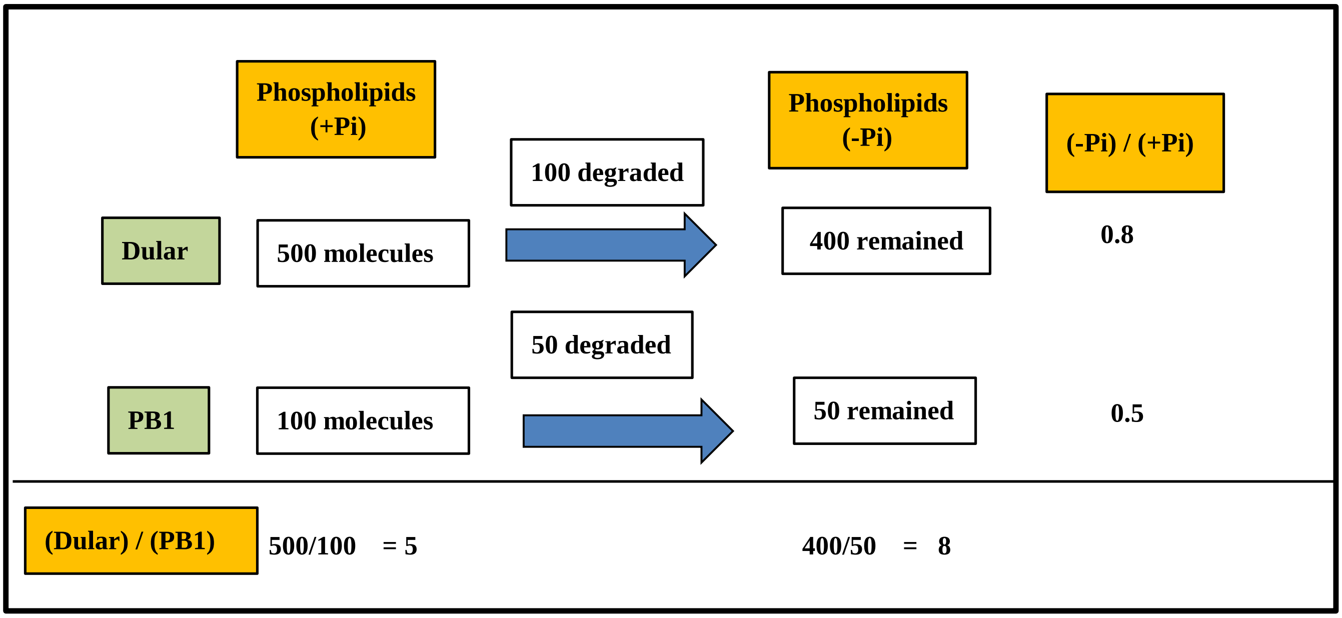

Supplementary Fig. S7 Analogical illustration of phospholipid dynamics in Dular and PB1. Suppose Dular has 500 starting molecules of phospholipid and PB1 have 100 molecules under Pi sufficient condition. Under low Pi condition these phospholipids are subjected to degradation. To estimate phospholipid degradation (-Pi/+Pi) was calculated. Ratios less than one means degradation of phospholipids in both genotypes under Pi deficiency. The rate of degradation is 50% for PB1 and 20% for Dular which reflects as PB1 is undergoing faster degradation. But more no. of absolute molecules are degraded in Dular (100) as compared to PB1 (50). This information can be retrieved indirectly by calculating ratio of phospholipid as Dular/ PB1. Higher ratio of Dular/PB1 (5) under Pi sufficient condition indicates higher phospholipid in Dular. Under -Pi, this ratio further increased because Dular after undergoing higher phospholipid degradation is still left with higher phospholipid reserve as compared to PB1. On the other hand PB1 has been depleted majority of phospholipids under low P condition which increases the ratio of Dular/PB1 (8).
